# Independent evolution of four heme peroxidase superfamilies

**DOI:** 10.1016/j.abb.2014.12.025

**Published:** 2015-05-15

**Authors:** Marcel Zámocký, Stefan Hofbauer, Irene Schaffner, Bernhard Gasselhuber, Andrea Nicolussi, Monika Soudi, Katharina F. Pirker, Paul G. Furtmüller, Christian Obinger

**Affiliations:** a Department of Chemistry, Division of Biochemistry, VIBT – Vienna Institute of BioTechnology, University of Natural Resources and Life Sciences, Muthgasse 18, A-1190 Vienna, Austria; b Institute of Molecular Biology, Slovak Academy of Sciences, Dúbravská cesta 21, SK-84551 Bratislava, Slovakia; c Department for Structural and Computational Biology, Max F. Perutz Laboratories, University of Vienna, A-1030 Vienna, Austria

**Keywords:** APx, ascorbate peroxidase, CcP, cytochrome *c* peroxidase, Cld, chlorite dismutase, DyP, dye-decolorizing peroxidase, EPO, eosinophil peroxidase, HGT, horizontal gene transfer, HRP, horseradish peroxidase, KatG, catalase–peroxidase, LDS, linoleate diol synthase, LPO, lactoperoxidase, LspPOX, *Lyngbya* peroxidase, ML, maximum likelihood method, MnP, manganese peroxidase, MPO, myeloperoxidase, PERCAL, calcium binding motif, PDB, Protein Data Bank, Pfam, protein families database, SCOP, structural classification of proteins, TPO, thyroid peroxidase, WSC, cell-wall integrity & stress response component, Heme peroxidase, Peroxidase–catalase superfamily, Peroxidase–cyclooxygenase superfamily, Peroxidase–chlorite dismutase superfamily, Peroxidase–peroxygenase superfamily

## Abstract

Four heme peroxidase superfamilies (peroxidase–catalase, peroxidase–cyclooxygenase, peroxidase–chlorite dismutase and peroxidase–peroxygenase superfamily) arose independently during evolution, which differ in overall fold, active site architecture and enzymatic activities. The redox cofactor is heme *b* or posttranslationally modified heme that is ligated by either histidine or cysteine. Heme peroxidases are found in all kingdoms of life and typically catalyze the one- and two-electron oxidation of a myriad of organic and inorganic substrates. In addition to this *peroxidatic* activity distinct (sub)families show pronounced catalase, cyclooxygenase, chlorite dismutase or peroxygenase activities. Here we describe the phylogeny of these four superfamilies and present the most important sequence signatures and active site architectures. The classification of families is described as well as important turning points in evolution. We show that at least three heme peroxidase superfamilies have ancient prokaryotic roots with several alternative ways of divergent evolution. In later evolutionary steps, they almost always produced highly evolved and specialized clades of peroxidases in eukaryotic kingdoms with a significant portion of such genes involved in coding various fusion proteins with novel physiological functions.

## Highlights

• Four heme peroxidase superfamilies arose independently during evolution.
• Description of their overall fold, active site architecture and typical sequence patterns.
• Report on phylogeny, unified classification and main enzymatic activities.
• Suggestion for consistent nomenclature within superfamilies.

## Graphical abstract

**Figure:**
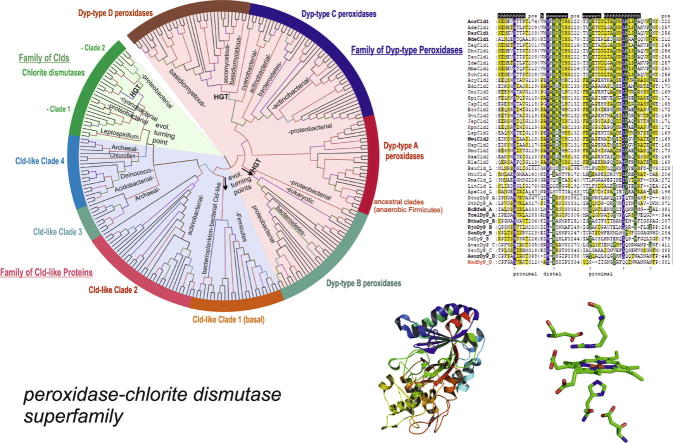

## Introduction

Heme peroxidases use heme *b* or posttranslationally modified heme as redox cofactor to catalyze the hydrogen peroxide-mediated one- and two-electron oxidation of a myriad of molecules including aromatic molecules (e.g., coniferyl alcohol or tyrosine), cations (e.g., Mn^2+^), anions (e.g., ascorbate or halides) or even proteins (e.g., cytochrome *c*). During turnover H_2_O_2_ is reduced to water and one-electron donors (AH_2_) are oxidized to the respective radicals (^•^AH) (Reaction 1) whereas two-electron donors like halides (X^−^) are oxidized to the corresponding hypohalous acids (HOX) (Reaction 2). Besides these *peroxidatic* reactions very few heme peroxidases also show a reasonable *catalatic* reaction (Reaction 3) and use a second hydrogen peroxide molecule as two-electron donor thereby releasing dioxygen. One additional activity catalyzed by a special group of heme peroxidases is the peroxygenation reaction, i.e., the (selective) introduction of peroxide-derived oxygen functionalities into organic molecules (Reaction 4).

(Reaction 1) $${\text{H}}_{2}{\text{O}}_{2}+2{\text{AH}}_{2}\to {\text{H}}_{2}\text{O}+2{}^{}\text{AH}$$

(Reaction 2) $${\text{H}}_{2}{\text{O}}_{2}+{\text{X}}^{-}+{\text{H}}^{+}\to {\text{H}}_{2}\text{O}+\text{HOX}$$

(Reaction 3) $${\text{H}}_{2}{\text{O}}_{2}+{\text{H}}_{2}{\text{O}}_{2}\to 2{\text{H}}_{2}\text{O}+{\text{O}}_{2}$$

(Reaction 4) $${\text{H}}_{2}{\text{O}}_{2}+\text{RH}\to {\text{H}}_{2}\text{O}+\text{ROH}$$

In the last decade – due to the application of powerful sequencing techniques – an ever increasing amount of protein sequences (including numerous heme peroxidases) were automatically assigned to related protein families due to typical conserved motifs [1]. However, critical analysis shows that these annotations often need corrections based on the knowledge of the relationship between sequence, structure and function of the respective protein family. An important aspect of sorting structural superfamilies is to classify the numerous sequences according to their phylogenetic relationships [2,3] and to identify turning points and evolutionary hybrids. Moreover, interesting newly discovered evolutionary clades frequently with yet unknown function and mostly marginal but important sequence variations can be selected for further structural and kinetic studies.

**Fig. 1:**
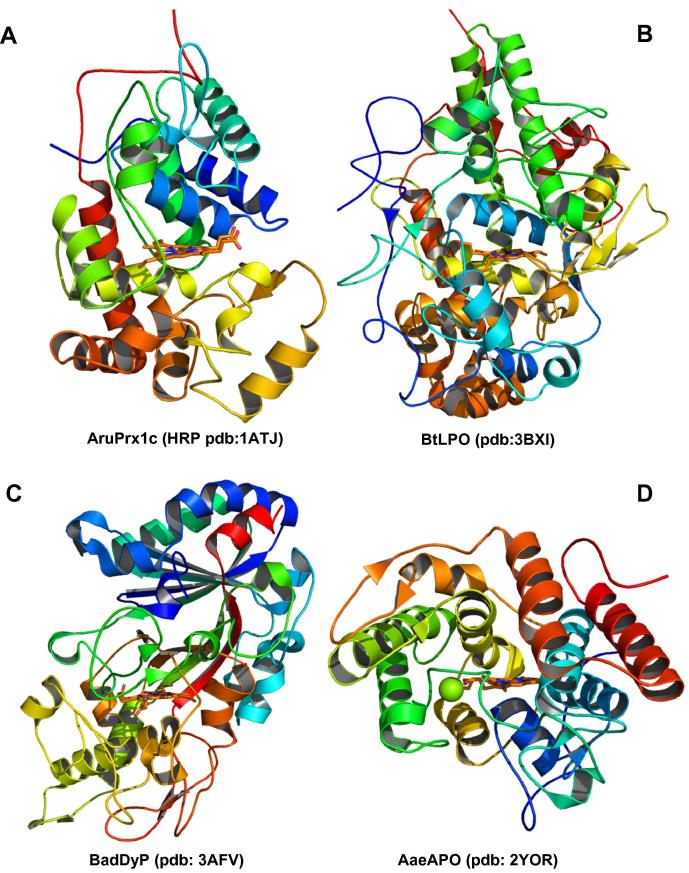
Structural fold typical for the peroxidase domains of each presented heme peroxidase superfamily. Representative structures with prosthetic heme group are shown for a typical member of (A) the peroxidase–catalase superfamily, (B) the peroxidase–cyclooxygenase superfamily, (C) the peroxidase–chlorite dismutase superfamily and (D) the peroxidase–peroxygenase superfamily. Abbreviations of presented peroxidases correspond with PeroxiBase and they are also explained in Supplem. Table 1.

This review demonstrates the independent evolution of four heme peroxidase superfamilies, namely of the (1) peroxidase–catalase superfamily, (2) peroxidase–cyclooxygenase superfamily, (3) peroxidase–chlorite dismutase superfamily and the (4) peroxidase–peroxygenase superfamily. We present an update of their reconstructed phylogeny and introduce their representative sequence signatures and essential amino acids in the heme cavity. From Fig. 1 it is obvious that each superfamily possesses a peculiar fold of the heme peroxidase domain that evolved independently with respect to other peroxidase superfamilies. It is further demonstrated that these ubiquitous oxidoreductases are present in all kingdoms of life. This strongly underlines the necessity to use the nomenclature suggested before (e.g., in [4] and not to talk about bacterial or plant or animal heme peroxidases as is still widespread in the literature and some databases). The denomination of the peroxidase superfamilies should reflect the characteristic enzymatic activities rather than their origin. The criteria for the definition of a peroxidase superfamily were presented already in [4] and here they are systematically applied on all four above listed heme peroxidase superfamilies that are further divided in particular families and subfamilies.

## Peroxidase–catalase superfamily

The peroxidase–catalase superfamily previously known as the “superfamily of bacterial, fungal and plant heme peroxidases” [5] is currently the most abundant peroxidase superfamily present in various gene and protein databases. Representatives of this superfamily were detected not only in the domains of Bacteria, Archaea, eukaryotic kingdoms of Fungi and Plantae but besides numerous Protozoa and Chromista species also within Metazoan kingdom [6]. In Pfam database (http://pfam.xfam.org) this universally distributed superfamily is annotated as PF00141.

**Fig. 2:**
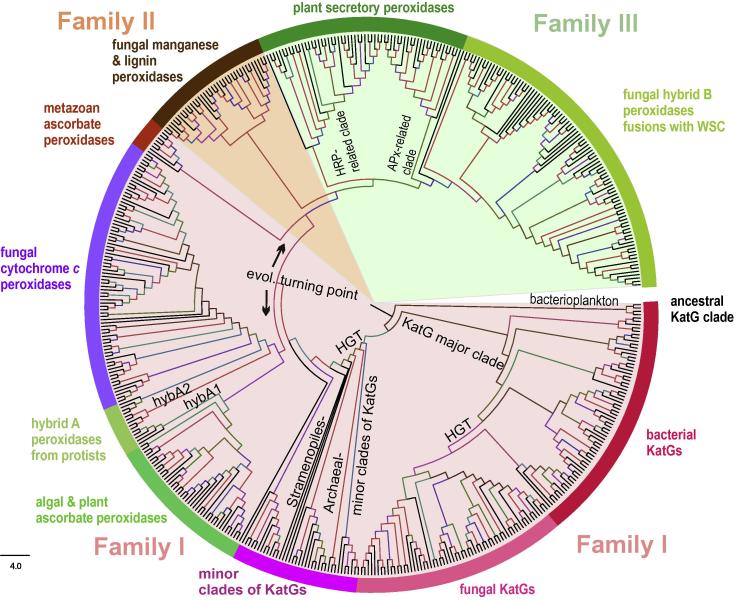
Reconstructed phylogenetic tree of 500 members of the peroxidase–catalase superfamily. The evolutionary history was inferred by using the maximum likelihood method based on the Whelan & Goldman model implemented in MEGA 5 software [66]. Bootstrap values are presented as color branches based on the obtained ML output: red > 90, violet > 70, blue > 50 and green > 30. All three main families (based on previously defined structural classes I, II and III known from [5]) are highlighted on the perimeter. Important subfamilies including the two hybrid-types are also highlighted.

**Fig. 3:**
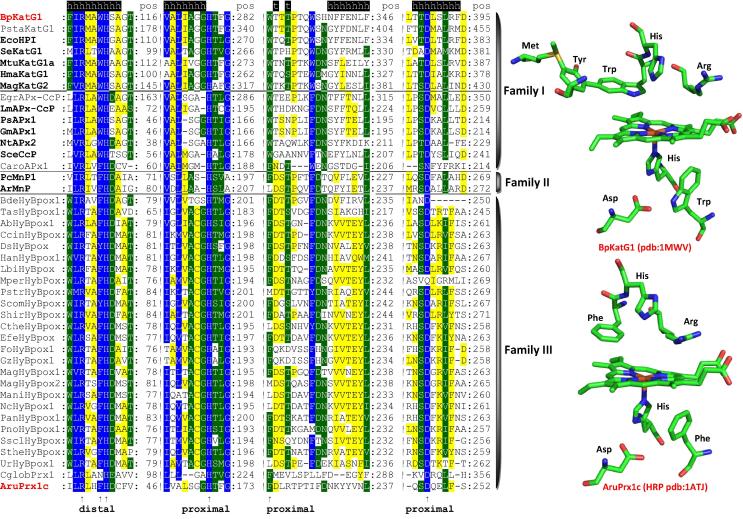
Typical sequence patterns including distal and proximal triad found in all representatives of the peroxidase–catalase superfamily. In addition, the active site architectures of the Family I member, catalase–peroxidase from *Burkholderia pseudomallei* (BpKatG1) and the Family III protein horseradish peroxidase (AruPrx1c) is depicted. Note that BpKatG1 shows the unique KatG-typical distal side adduct that includes the distal tryptophan. Abbreviations of sequence names are explained in Supplem. Table 1 and they correspond with the nomenclature of PeroxiBase (http://peroxibase.toulouse.inra.fr). Color scheme: blue highest sequence similarity (93% conservation), green moderate similarity (75%), yellow low similarity (33%). Secondary structure elements for BpKatG1 structure are given above the alignment.

Here we have selected 500 representative full-length sequences that were used for an updated phylogenetic reconstruction based on the maximum likelihood (ML) method. Fig. 2 shows the occurrence of three well separated families already defined by K. Welinder in 1992 as distinct structural classes Class I, II and III [5]. We suggest to use for them the term *families* in analogy with all other heme peroxidase superfamilies (see below). Novel clades representing hybrid enzymes between the previously defined classes are well distinguishable in this reconstruction. Fig. 3 shows the typical sequence signatures of this superfamily that includes the distal amino acid triad Arg-Trp/Phe-His within the sequence –X-**R**-XX-**W**/**F**-**H**-X- and the proximal triad His-Trp/Phe-Asp (these amino acids are not neighbored in the primary sequence). The typical overall globular fold of representatives of this superfamily consists of twelve α-helices and was already acquired from the beginning. It was only slightly modified in the later steps of ongoing divergent evolution. Generally, the level of structural conservation is higher than the conservation of amino acid sequences [7].

Family I was previously defined to contain intracellular bacterial catalase–peroxidases [8,9], cytosolic, chloroplastic and peroxisomal ascorbate peroxidases [10] and mitochondrial (membrane bound) cytochrome *c* peroxidases [11,12]. Catalase–peroxidases are the only representatives of this superfamily with a high *catalatic* activity according to Reaction 3 [13] besides a peroxidatic activity. Ascorbate and cytochrome *c* peroxidases are typical monofunctional peroxidases (i.e., cannot catalyze Reaction 3) that follow Reaction 1 with either ascorbate or cytochrome *c* as one electron donor (AH_2_). The main function of Family I peroxidases seems to be scavenging of excess H_2_O_2_.

Family I is the most divergent one among the three structural classes. It contains eukaryotic intracellular [14,15] or even peroxisomal [16] as well as extracellular [17] catalase–peroxidases and also putative cytochrome *c* peroxidases from Choanoflagellida predecessors of fungi and animals [6]. Moreover, hybrid-type A heme peroxidases were shown to belong to Family I [7]. Their biochemical properties are between those of monofunctional APxs and CcPs [18,19], thus they represent an important turning point from ancient bifunctional catalase–peroxidases towards monofunctional specialization within this large superfamily (Fig. 2).

A peculiar minor descendant clade containing yet putative secretable ascorbate peroxidases from Unikonts/Metazoan lineages represents another evolutionary turning point (Fig. 2). A majority of further evolutionary descendants of this clade were verified as extracellular secreted proteins [6]. Among Family II sequences well known and intensively investigated lignin, manganese, versatile and generic peroxidases are found. Their main function seems to be the degradation of lignin-containing soil debris [20,21]. Recently, several novel clades with putative Family II members were detected also in various ascomycetous fungi unable to decay wood (Fig. 2). Thus, their physiological function and substrate specificity remain to be elucidated. Principally, they are able to catalyze Reactions 1 and 2.

The highest number of members are found in Family III that is comprised of numerous plant secretory peroxidases like horseradish peroxidase(s) (HRP) [e.g., 22] but also by a parallel abundant clade divided into “APx-related” yet putative algal and plant peroxidases [23] and hybrid-type B peroxidases that are present solely in the kingdom of fungi [6,7]. Sequence analysis has shown that most of these hybrid-type B peroxidases contain an additional C-terminal WSC sugar binding domain of unknown function. Family III peroxidases mainly follow Reaction 1. They are generally secreted into the cell wall or the apoplastic compartment and the vacuole and play a role in the oxidation of lignin precursors, auxin and secondary metabolites [22].

## Peroxidase–cyclooxygenase superfamily

**Fig. 4:**
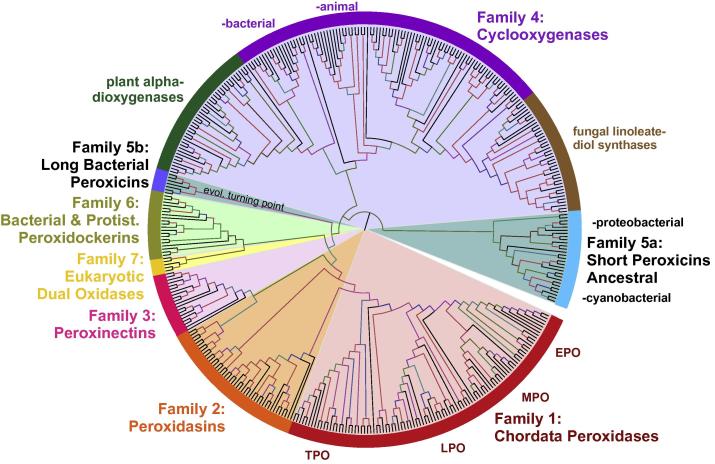
Reconstructed phylogenetic tree of 400 members of the peroxidase–cyclooxygenase superfamily. The evolutionary history was inferred by using the maximum likelihood method based on the Whelan & Goldman model implemented in MEGA 5 software [66]. Bootstrap values are presented as a color branches based on the ML output: red > 90, violet > 70, blue > 50 and green > 30. All seven (numbered) families known from [4] are highlighted on the perimeter together with important subfamilies.

The peroxidase–cyclooxygenase superfamily has Pfam accession PF03098 and its members are widely distributed among all domains of life [4]. Therefore, its (old) denomination as “animal heme-dependent peroxidases” is misleading but still present in some public databases. We have selected 400 representative sequences for an updated ML phylogenetic reconstruction (Fig. 4). The seven main families are well conserved in this update but some of them are presented as more abundant if compared with previous reconstructed phylogenies [24–26] that focused on particular (sub) families.

**Fig. 5:**
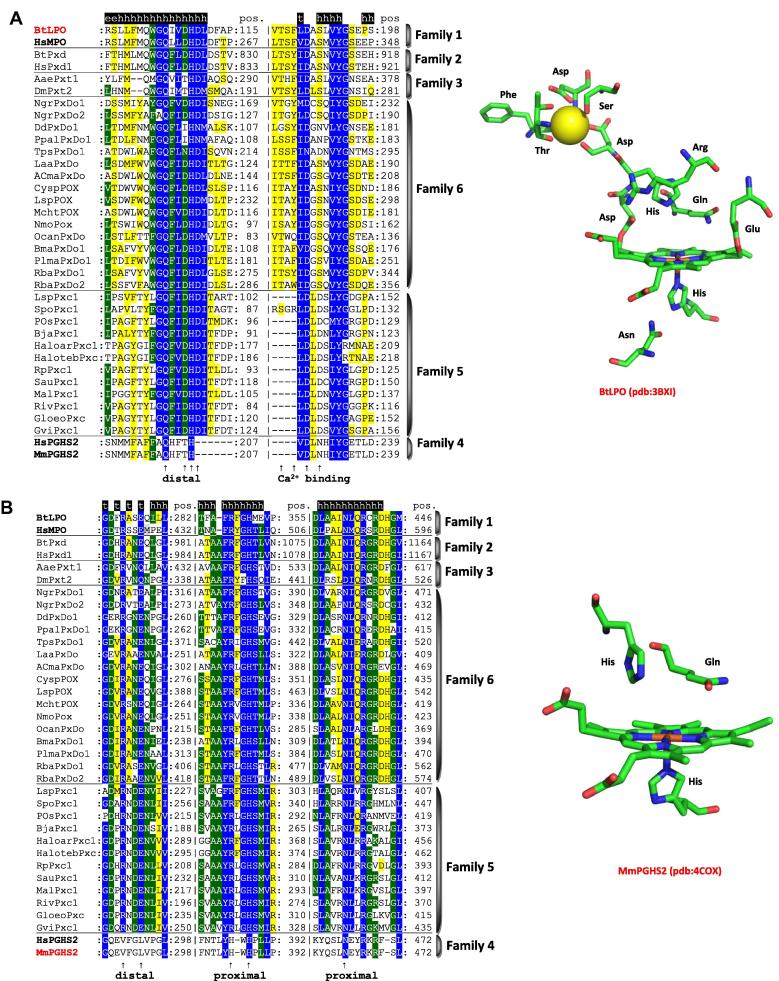
Typical sequence pattern including distal and proximal residues found in representatives of the peroxidase–cyclooxygenase superfamily (except dual oxidases that were shown to contain a mutated peroxidase domain without peroxidase activity [39]. (A) Distal heme side and calcium binding motif, (B) further distal motif and proximal heme side. Color scheme: blue highest similarity (93% conservation), green moderate similarity (70%), yellow low similarity (33%). In addition the active site architectures of bovine lactoperoxidase and mouse prostaglandin H synthase 2 are depicted. Abbreviations of sequence names are explained in Supplem. Table 1 and they correspond with the nomenclature of PeroxiBase (http://peroxibase.toulouse.inra.fr). Secondary structure elements for BtLPO structure are given above the alignment.

In contrast to the peroxidase–catalase superfamily, in many cases members of the peroxidase–cyclooxygenase superfamily are multidomain proteins with one heme peroxidase domain of predominantly α-helical fold with a central heme-containing core of five α-helices. Moreover, this superfamily is unique in having the prosthetic heme group posttranslationally modified [26,27]. The heme is covalently bound to the protein via two ester linkages formed by conserved Asp and Glu residues (Fig. 5A). In one representative (i.e., myeloperoxidase) a third heme to protein linkage is formed [28]. As a consequence of these modifications the heme is distorted and these peroxidases exhibit unique spectral and redox properties [29]. All representatives catalyze both Reactions 1 and 2 but with most members studied so far halide oxidation seems to be the dominating physiological enzymatic activity.

Fig. 5A and B show the typical sequence signatures that allow the correct assignment of peroxidases to this superfamily. Except for families 4 and 7 the most typical sequence is -X-G-**Q**-X-X-**D**-**H**-**D**-X- which includes the distal catalytic histidine that is neighbored by two aspartates, the first being involved in ester bond formation and the second in Ca^2+^-binding. The highly conserved glutamine seems to be involved in halide binding [30] (Reaction 2). Fig. 5A shows the active site of lactoperoxidase, which is a well studied member of family 1. Further typical distal residues include the catalytic arginine and a conserved glutamate that is involved in formation of the second ester bond. The typical sequence is -X-**R**-X-X-**E**-X- (Fig. 5B). The proximal ligand of peroxidases from the peroxidase–cyclooxygenase superfamily is a histidine hydrogen bonded to an asparagine (except Family 4 & 7) (Fig. 5B). Additionally, the calcium binding motif appears to be not fully conserved among the ancestral members (Fig. 5). It is conserved in families 1–3.

The ancestral clade of short bacterial peroxicins (i.e., family 5) is supposed to contain the oldest representatives of this superfamily (Fig. 4), namely the short peroxicins of cyanobacteria *Gloeobacter violaceus*, *Rivularia* sp. and *Gloeocapsa* sp. which might represent (as molecular fossils) the early evolved heme peroxidases to cope with reactive oxygen species produced by ancient oxygenic photosynthesis [31]. From them, further proteobacterial and actinobacterial short peroxicins evolved (Fig. 4). Their closest descendant branch of long bacterial peroxicins represents a real turning point in the early phase of evolution within this superfamily. Possibly through several gene duplications and subsequent fusion events very large open reading frames (theoretically from 1600 up to 3300 amino acids) emerged in proteobacterial genomes. In such long ORFs, not only the peroxidase domains were duplicated (and fused) but also two different calcium binding motifs, namely PERCAL and hemolysin-type occur repeatedly [32]. Although the expression of such multi-domain proteins could not be verified yet, these long fusions were probably the basis for intensive further molecular evolution that proceeded in two quite different directions. The physiological role of Family 5 peroxidases that contains in the current reconstruction only putative sequences is completely unknown.

One evolutionary trend led to the emergence of the well known animal peroxidases like myeloperoxidase (MPO), eosinophil peroxidase (EPO), lactoperoxidase (LPO) and thyroid peroxidase (TPO) and started with bacterial peroxidockerins (Family 6). The cyanobacterial LspPOX is a typical representative of early stage in this direction and it was already investigated on protein level [26,33].

The second evolutionary direction leads towards bacterial and animal cyclooxygenases (Family 4) (Fig. 4). Although bacterial cyclooxygenases are still at the level of putative protein sequences, several cyclooxygenases from the animal phyla Cnidaria, Mollusca, Arthropoda and Chordata were already cloned and analyzed [34]. Multiple paralogs of this family have arisen from repeated gene duplication events and are separately regulated in each organism. A very important and peculiar branch of this family is presented by plant alpha dioxygenases that diverged early from cyclooxygenases (Fig. 4). Although they retained the conserved predominantly α-helical fold of this superfamily [35] slight structural modifications lead to decreased peroxidase activity whereas oxygenation of fatty acids to corresponding 2(R)-hydroperoxides is the main physiological activity of these enzymes [36]. In the opposite direction of this family evolution, we can observe a very abundant subfamily of fungal linoleate diol synthases (LDS) that apparently evolved from cyclooxygenases (Fig. 4). A few ascomycetous representatives were already purified and investigated [37,38] revealing a fusion of N-terminal peroxidase domain with C-terminal P450 domain. By studying the corresponding deletion mutants, the regulation of LDS through protein kinase A mediated pathway was detected [38].

The alternative evolutionary trend leading from long peroxicins towards Families 3–1 occurred stepwise. First steps led over (already above mentioned) peroxidockerins (Family 6). It is important to note that evolutionary descendants of variable length emerged not only in eubacteria but can be also found in genomes of eukaryotic Amoebozoa, Heterolobosea and Chromalveolata (Fig. 4). This fact indicates that peroxidockerins were widely distributed among the early eukaryotic world but whether there was one or several HGTs from marine bacteria towards unicellular ancestral eukaryotes remains to be elucidated in the future research if more sequences of this Family 6 will be made available.

Dual oxidases (Family 7) were segregated from peroxidockerins at the level of primitive eukaryotes as stated before ([4] and Fig. 4). These multidomain oxidases have retained an extracellular peroxidase domain at the N-terminus followed by a transmembrane segment appended to an EF-hand calcium-binding cytosolic region and a cytosolic C-terminal enzymatic domain with homology to NADPH oxidase. The peroxidase domain is significantly mutated (proximal histidine is absent) and lost its peroxidase activity [39]. It was shown that dual oxidases generate H_2_O_2_ but the function of the modified peroxidase domain in this large multidomain protein remains obscure [40].

In a further step peroxinectins (Family 3) evolved. They were shown to exhibit cell adhesion function(s) and to be involved in invertebrate immune response by production of hypohalous acids according to Reaction 2 [41]. In principle, they are also fusion proteins of a conserved heme peroxidase domain with an integrin-binding motif (i.e., KGD [41]) that probably co-evolved from the dockerin part of peroxidockerins together with the peroxidase domain. Peroxinectins are widely spread mainly among the Eumetazoan phyla arthropods and nematodes with occurrence of several paralogs in a single genome as result of frequent gene duplications.

Next evolutionary event led to division in peroxidasins (Family 2) and a sister family of Chordata peroxidases (Family 1). They diverged in several Chordata subfamilies including the separated subfamily of thyroid peroxidases, minor subfamilies of fish peroxidases and three closely connected subfamilies of mammalian lactoperoxidases (LPO), myeloperoxidases (MPO) and eosinophil peroxidases (EPO) (Fig. 4) that are major players in the innate immune system by the production of antimicrobial hypohalous acids [30]. The overall phylogenetic distribution of peroxidase orthologs in four mammalian monophyletic clades (i.e., subfamilies) is in accordance with the general evolution and speciation of these organisms with a positive selection detected at specific amino acid sites for slight functional shifts [42] with impact on innate immunity. Thyroid peroxidase (TPO) catalyzes the formation of thyroid hormones in mammals [43].

In contrast, we can observe a broad and rather abundant distribution of peroxidasin isozymes in various Eumetazoan phyla (Fig. 4 and [25]). They were segregated in one minor subfamily of (yet putative) short peroxidasins and up to five distinct subfamilies of long (multi-domain) peroxidasins where the peroxidase domain is fused N-terminally with domains of leucine-rich repeats and immunoglobulin type-C whereas C-terminally with von Willebrand factor C module. The subfamilies of long peroxidasins apparently evolved through many speciation events from Urochordata to Mammals [25]. The physiological function of peroxidasins was comprehensively studied only recently [44]. Besides expected (unspecific) antimicrobial activity through production of hypohalous acids (common with other chordata peroxidases) they play an important role in the biosynthesis of extracellular matrix. Human peroxidasin 1 uses Reaction 2 to catalyze specifically the essential formation of sulfilimine bonds in collagen IV thus being involved in the biosynthesis of the extracellular matrix [45]. Cross-linked collagen IV scaffold is essential for epithelial tissue genesis and stabilization. Systematic analysis showed that both collagen IV crosslink formation as well as tissue-specific peroxidasin evolution probably arose at the point of divergence between Metazoan phyla Porifera and Cnidaria as evolutionary innovation for a specialized extracellular matrix–basement membrane of animal tissues [44].

## Peroxidase–chlorite dismutase superfamily

**Fig. 6:**
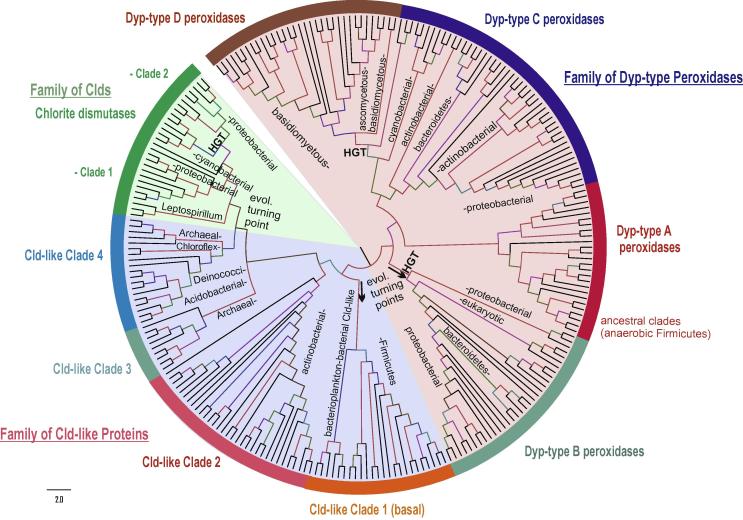
Reconstructed phylogenetic tree of 250 members of the peroxidase–chlorite dismutase superfamily. The evolutionary history was inferred by using the maximum likelihood method based on the Whelan & Goldman model implemented in MEGA 5 software [66]. Bootstrap values are presented as color branches based on the ML output: red > 90, violet > 70, blue > 50 and green > 30. Three main families [chlorite dismutases (Clds), dye-decolorizing peroxidases (DyPs) and Cld-like proteins] known from [46,49] are highlighted on the perimeter. Important subfamilies (in some cases clades) are also shown.

This superfamily was first constituted by Goblirsch et al. [46] bringing together related sequences of three distinct protein families that share a common fold (however with low overall sequence identity) and may possess essential and related functions in iron-dependent metabolism. A common heme-binding scaffold of so-called dye-decolorizing peroxidases (DyPs) with chlorite dismutases (Cld) and their evolutionary intermediates (i.e., deferrochelatase/peroxidases and Cld-like proteins) was the most important prerequisite for defining this novel superfamily of heme proteins (Fig. 6) [46]. Originally, this superfamily was designated as CDE (i.e., chlorite dismutase-DyP–EfeB) superfamily [46]. Updated phylogeny demonstrates that EfeB belongs to DyPs.

**Fig. 7:**
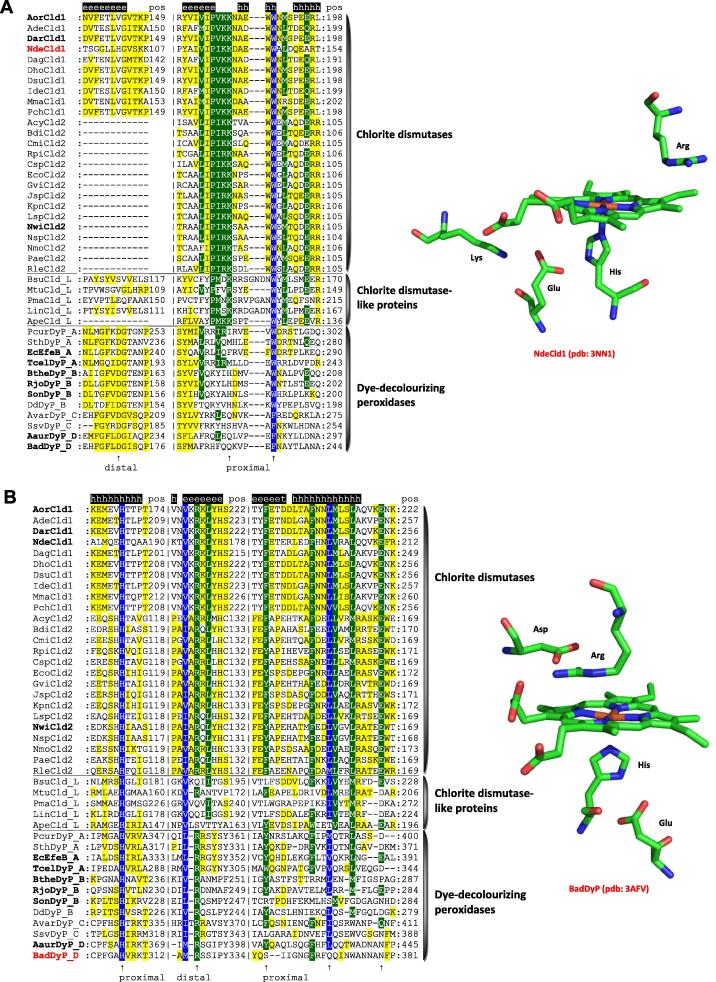
Typical sequence pattern including distal and proximal residues found in representatives of the peroxidase–chlorite dismutase superfamily. Chlorite dismutases (Clds), dye-decolorizing peroxidases (DyPs) and Cld-like proteins. Color scheme: blue highest similarity (93% conservation), green moderate similarity (67%), yellow low similarity (30%). In addition the active site architectures of chlorite dismutase from *Nitrospira defluvii* (Clade 1 Cld) and of DyP from *Bjerkandera adusta* (Dyp-type D) are depicted. Abbreviations of sequence names are explained in Supplem. Table 1 and for peroxidase representatives correspond with the nomenclature of PeroxiBase (http://peroxibase.toulouse.inra.fr). Secondary structure elements for NdeCld1 structure are given above the alignment.

Fig. 7 depicts typical sequence patterns and heme cavity architectures of DyPs and Clds. The dye-decolorizing peroxidases and Clds show a fully conserved arginine at the distal heme site [47]. DyPs in addition have a catalytic aspartate, whereas in Cld-like proteins both residues are missing. In all members of the peroxidase–chlorite dismutase superfamily the proximal ligand is a histidine, which in Clds is hydrogen bonded to a glutamate, in DyPs to an aspartate. Another Cld-typical sequence pattern is -X-**I**-**P**-**V**/**I**-**K**/**R**-**K**-X- that includes proximal residues (Fig. 7).

Although the particular families possess two different accessions in Pfam, namely PF04261 (for DyP) and PF06778 (for Cld), they are classified in a common protein clan CL0032 (SCOP 54909) that comprises up to 18 member families, but most of them without the ability to bind the prosthetic heme group. It is important to note that in contrast to the two previously described heme peroxidase superfamilies, the peroxidase–chlorite dismutase family appears to cover only a small part of a very large dimeric α/β barrel structural superfamily (i.e., protein clan). The modification of such a fold to acquire the ability to bind the heme group and exploit it evolved probably only in one evolutionary event. The whole dimeric α/β barrel structural superfamily is outside of the scope of this issue.

DyP heme peroxidases are abundant not only in many bacterial phyla [48] but also among fungi. Recently a partial phylogeny focussing on 160 fungal sequences was reconstructed [20]. Further phylogenies comprising also Cld sequences [46,49] revealed the extent and diversity of these peculiar heme peroxidases. Our updated phylogenetic reconstruction covering all taxonomical divisions is shown in Fig. 6. In principle, we can distinguish four DyP subfamilies known from previous classification [50,51], four Cld-like clades (i.e., subfamilies to be further resolved in the future) and two clades of functional Clds (Fig. 6). Dye-decolorizing peroxidases mainly follow Reaction 1 and exhibit a very broad substrate range. Originally, they were found to degrade bulky dyes and from these observations the denomination dye-decolorizing peroxidase derived. Functional Clds are able to degrade chlorite efficiently to chloride and O_2_, whereas Cld-like enzymes neither exhibit a peroxidase nor a chlorite degrading activity. Moreover, Cld-like proteins show a diminished affinity for the prosthetic group.

The ancestral clade of the whole superfamily is probably located in DyP-subfamily A, namely among sequences from thermophilic facultatively anaerobic bacteria of the division Firmicutes (Fig. 6). This would suggest ancient roots of the whole superfamily as thermophilic anaerobic bacilli are supposed to be of very old origin [52]. From this ancestral DyP-A subfamily the evolution led either to more sophisticated DyP-C and DyP-D subfamilies or in alternative direction towards shortened DyP-subfamily B and then further to subfamilies of Clds. DyP-type C & D subfamilies cluster together with a high bootstrap support but whereas DyP-C representatives are known from genomes of proteobacteria, actinobacteria and cyanobacteria, DyP-D members are only present among basidiomycetous and ascomycetous fungi. Thus, DyP-type D peroxidases from the fungal subkingdom of Dikarya apparently evolved via horizontal gene transfer (HGT) from cyanobacterial predecessors (see Fig. 6) that belong to the DyP-type C subfamily. Blast searches in genomes of ancestral (early diverging) fungi like *Mucor circinelloides* (a Zygomycete that contains also genes from the peroxidase–peroxygenase superfamily, see below) did not yield any output for DyP peroxidase genes. This supports the theory of HGT from autotrophic prokaryotes to heterotrophic eukaryotes. Anyway, upcoming new genomes of early diverging fungi must be also screened with blast in this respect. Their codon usage has to be analyzed and compared.

Fungal DyP sequences occur not only in subfamily D but also in subfamily B, where also several other sequences from lower eukaryotes like *Dictyostelium discoideum* or *Naegleria gruberi* DyP-peroxidases occur. Most of here presented protein sequences from this subfamily stem from Proteobacteria therefore also in subfamily B HGT events appeared probably several times during the evolution. Even more surprising is the occurrence of DyP-type B genes in the Trematode parasite *Schistosoma mansoni*. Also in this multicellular flatworm DyP genes were acquired by HGT from lower eukaryotes but their functionality must be proven experimentally. Thus it appears that subfamily B is the most divergent one of all four described DyP subfamilies leading to unique evolution of DyP peroxidases within Unicont genomes. Turning points in this superfamily’s evolution lie unequivocally in this subfamily clades that besides several HGT events enabled also further evolution towards chlorite-dismutase like proteins.

Among four distinct Cld-like subfamilies (Fig. 6) the clade containing mainly Firmicutes representatives but also several archaeal sequences appears to be basal for further evolution towards functional chlorite dismutases. It is important to note that within archaeal genomes only Cld-like sequences were found yet but no sequences coding for DyPs. This underlines the hypothesis that Clds evolved in a later phase of the diversification from DyP B subfamily (Fig. 6) via shortening of the gene. But as both DyPs and Clds are present as paralogs in genomes of extant Cyanobacteria, Firmicutes or Proteobacteria, Cld (or Cld-like) genes must have been transferred in mentioned bacteria by the means of secondary HGTs in later stages of this superfamily’s evolution. The stepwise natural mutations in Cld-like clades finally led to two functional clades (i.e., subfamilies) of chlorite dismutases as probably the latest step of this long-term evolution. Whereas the physiological function of numerous putative Cld-like proteins is under discussion [58–60], the fully functional chlorite dismutases containing heme *b* prosthetic group are segregated in two distinct clades (6). Clade 1 is almost completely formed by proteobacterial penta- or hexameric representatives whereas Clade 2 contains dimeric proteobacterial as well as numerous cyanobacterial enzymes. The N-terminus of Clade 2 proteins is truncated (Fig. 7) [49]. Some of them were investigated recently on protein level [53–57]. The question remains, however, whether removal of chlorite is indeed their native physiological function as higher concentrations of chlorite most likely occur in the environment just from anthropogenic actions of the last 50 years [46].

Observed conserved and variable patterns from the multiple sequence alignment support the above formulated phylogenetic hypothesis that Clds evolved from DyP-B peroxidases by partial deletions and modifications within the frequently but not stringently duplicated ancestral DyP-related gene. Some of the Cld-like clades that have lost the essential functional residues may represent “death-end products” of superfamily evolution (seen also in peroxidase–cyclooxygenase superfamily [4]) or they have acquired some new functions (e.g., in heme biosynthesis [58–60] that were different from the ancestral peroxidase activity.

## Peroxidase–peroxygenase superfamily

This heme peroxidase superfamily is unique since its members can (besides typical *peroxidatic* activity) efficiently incorporate peroxide-derived oxygen into substrate molecules. Additionally their proximal heme ligand is cysteine instead of histidine (Fig. 9). In the literature several names are used for its members including heme-thiolate peroxidases (HTPs), unspecific peroxygenases (UPOs) or aromatic peroxygenases (APOs). For several decades chloroperoxidase from the ascomycetous fungus *Leptoxyphium* (*Caldariomyces*) *fumago* has been in the focus of research only. In the last decade other members of this superfamily (Pfam accession number PF01328) were found and started to be analyzed.

**Fig. 8:**
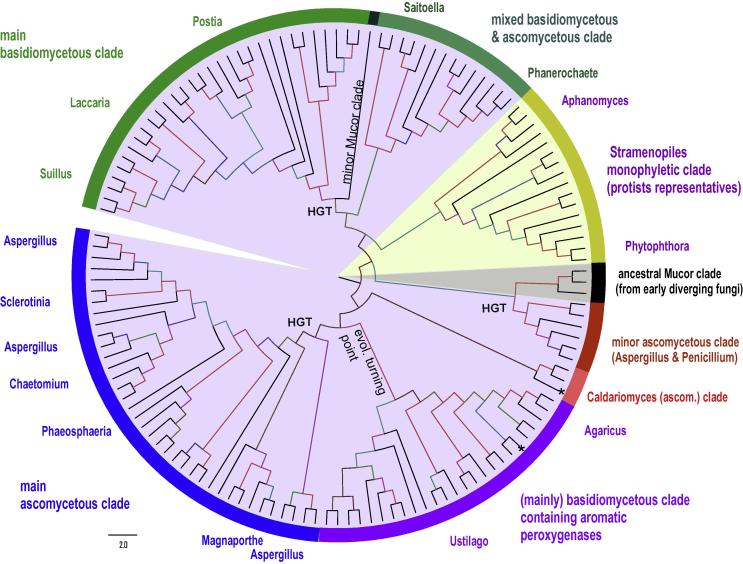
Reconstructed phylogenetic tree of 136 members of the peroxidase–peroxygenase superfamily. The evolutionary history was inferred by using the maximum likelihood method based on the Whelan & Goldman model implemented in MEGA 5 software [66]. Bootstrap values are presented as color branches based on the ML output: red > 90, violet > 70, blue > 50 and green > 30. All main subfamilies (currently defined only as clades) are highlighted on the perimeter.

From the beginning of their discovery it was clear that these oxidoreductases differ from all previously known heme peroxidases and are functional hybrids between a “classical” heme peroxidase and a P450-monooxygenase [61]. These metalloproteins are secreted and highly glycosylated versatile enzymes that catalyze the oxidation of organic heteroatoms and inorganic halides, one-electron oxidations (Reactions 1 and 2) but also hydroxylations (Reaction 4), epoxidations and dealkylations [62–64]. Therefore, we suggest the denomination peroxidase–peroxygenase superfamily. Its members show a typical fold with ten α-helices and five very short β-sheets [61,65]. Currently, it is the smallest known heme peroxidase superfamily but its full phylogenomic extent still has to be discovered. This means that the particular families cannot be clearly defined yet and the current phylogenetic reconstruction (Fig. 8) comprises only division in main clades. A partial phylogeny was already reconstructed for isolates of forest floor samples [20] that focused predominantly on basidiomycetous sequences. Here, we show an updated phylogeny based on 136 representatively selected sequences that cover all organisms with already detected heme-thiolate peroxidase genes (Fig. 8) most of them still putative.

**Fig. 9:**
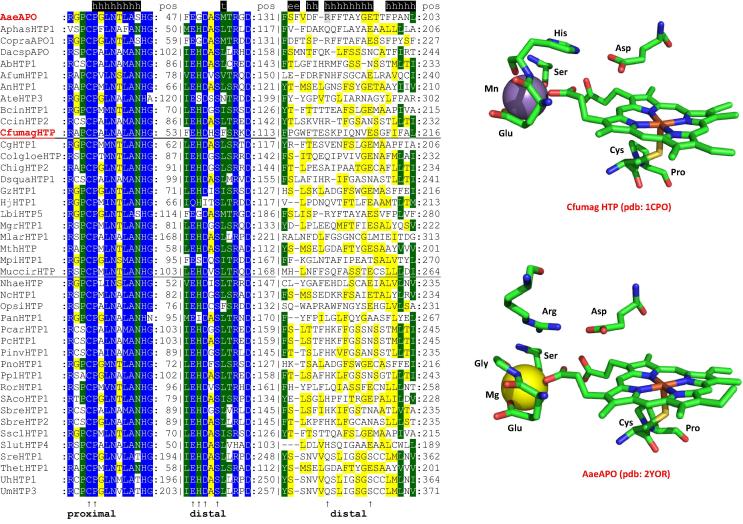
Typical sequence pattern including distal and proximal residues found in representatives of the peroxidase–peroxygenase superfamily. Color scheme: blue highest similarity (93% conservation), green moderate similarity (67%), yellow low similarity (33%). In addition the active site architectures of enzymes from *Caldariomyces fumago* and *Agrocybe aegerita* are depicted. Note that both proteins have a cation-binding site in the heme periphery. Abbreviations of sequence names are explained in Supplem. Table 1 and they correspond with the nomenclature of PeroxiBase (http://peroxibase.toulouse.inra.fr). Secondary structure elements for AaeAPO structure are given above the alignment.

Typical conserved regions of members of this superfamily show the -X-**P**-**C**-**P**-X- motif which includes the proximal heme ligand cysteine and the -X-**E**-**G**-**D**-X- motif that contains three ligands involved in the binding of a cation (Fig. 9). Interestingly, the distal side, where the enzymatic reaction occurs, shows a surprising variability. In those representatives with an experimentally verified peroxidase and peroxygenase reaction an aspartate together with a basic amino acid is found (Fig. 9).

The ancestral clade appears to contain *Mucor* enzymes as representatives of genes from early diverging fungi of the division Zygomycota. This means that those genes were – in contrast with some other peroxidase subfamilies (e.g., Dyp-D or -B see above) – included in the genomes of ancestral, primitive fungi already from the beginning of the evolution of the fungal kingdom. The monophyletic clade of Stramenopiles enzymes diverged apparently very early in parallel with the evolution of “classical” but minor fungal enzymes represented by CfuHalPrx from the ascomycete *Caldariomyces fumago* with known 3D structure [61] and reactivity investigated in detail [63,64]. Putative Stramenopiles sequences currently represent the only monophyletic clade of “non-fungal” heme-thiolate peroxidases. Whether there are other related genes present in further protistan genomes remains to be elucidated but until now no such sequences were found. In contrast with early diverging ascomycetous lineages, unspecific aromatic peroxygenases like those from basidiomyceta *Auricularia delicata* or *Coprinellus radians* are results of later evolutionary events from *Mucor* peroxidases via repeated gene duplication and speciation events (Fig. 8). The development of the minor clade containing mainly sequences from various mushrooms can be considered as evolutionary turning point because the properties of the typical member – *Auricularia* heme-thiolate peroxidase (AaeAPO) reveal slight differences in the heme pocket if compared to CfumagHTP [65]. The main ascomycetous clade, as well as the mixed basidiomycetous and ascomycetous clade and the main basidiomycetous clade contain numerous representatives that are completely putative at the moment. With the exception of the second minor *Mucor* clade no sequences from phyla other than dikaryotic fungal subkingdom are known in the four above mentioned abundant clades yet. As most here presented sequences are only putative, the future research shall uncover their peculiar enzymatic properties not only for their peroxidatic activity but for oxyfunctionalization of organic molecules in general [64]. Some of these sequences are found in genomes of pathogenic fungi so their putative roles as secretable enzymes in pathogenesis and host–pathogen interactions have to be evaluated. Nevertheless, it is currently unclear how this peroxidase–peroxygenase superfamily could have evolved from more primitive forms. Search in various (gene and protein) databases reveals that it shows the lowest abundance among here described families of heme containing peroxidases.

## Appendix A Supplementary data

Supplementary data 1 This Supplementary material contains Supplem. Table 1.
